# Bruton's Tyrosine Kinase: A Promising Target for the Treatment of COVID-19

**Published:** 2020-11

**Authors:** Mitra Rezaei, Abdolreza Babamahmoodi, Majid Marjani

**Affiliations:** 1 Virology Research Center, National Research Institute of Tuberculosis and Lung diseases (NRITLD), Shahid Beheshti University of Medical Sciences, Tehran, Iran,; 2 Clinical Tuberculosis and Epidemiology Research Center, NRITLD, Shahid Beheshti University of Medical Sciences, Tehran, Iran

## Dear Editor,

Coronavirus Disease 2019 (COVID-19) caused by the severe acute respiratory syndrome coronavirus 2 (SARS-CoV-2) emerged in December 2019 in China and quickly spread throughout the world. By June 24, 2020, the World Health Organization (WHO) reported the total number of 8,993,659 laboratory-confirmed cases leading to 469,587 deaths worldwide (1). COVID-19 has a range of clinical manifestations from asymptomatic cases to severe and life-threatening illnesses (2). The most severe form of the disease is the rapidly progressing failure of the respiratory system, presenting by severe dyspnea and profound hypoxemia, and may lead to acute respiratory distress syndrome (ARDS)(3) .

The pathophysiology of COVID-19 is under investigation and has not yet certainly defined. Like other severe forms of coronavirus diseases, the hyperactivation of the immune system resulting in hyper inflammation and cytokine storm syndrome has been postulated (4) . Most of the patients with the severe form of COVID-19 have higher serum levels of various inflammatory cytokines and chemokines, including interleukin-1β (IL-1β), IL-6, granulocyte colony-stimulating factor (G-CSF), granulocyte/macrophage colony-stimulating factor (GM-CSF), interferon-γ (IFN-γ), tumor necrosis factor (TNF), and macrophage inflammatory protein-1α (MIP1α)(5, 6) .

Although more than 350 ongoing clinical trials concerning potentiality effective therapeutic agents are underway, unfortunately, no treatment has been proved to treat COVID-19 cases (7). Due to some similarities between COVID-19 and macrophage activation syndrome, targeting the innate immune system may be an effective strategy to control the disease (8).

**Figure 1.**
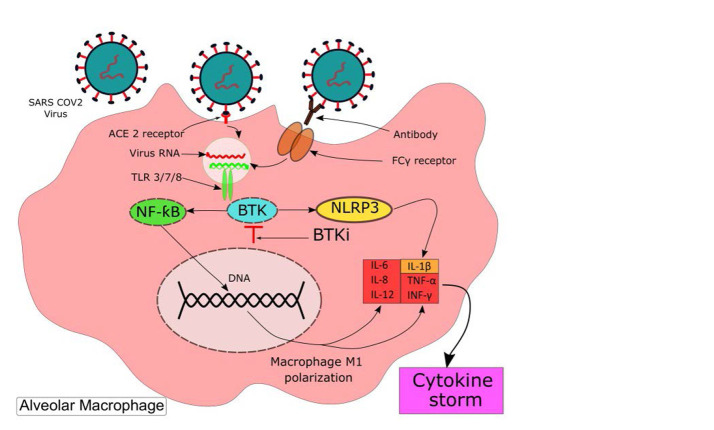
BTK-dependent signaling in alveolar macrophage and its proposed role in cytokine storm In comparison with alveolar cells, the ACE_2_ receptor is expressed at a lower extent on the macrophage surface. Phagocytosis of virus-containing immune complexes via FcγRs is the principal mechanism of macrophage infection (19). TLRs recognize the virus RNA and initiate signaling through BTK-dependent activation of NF-ƙB resulting in the production of inflammatory cytokines and chemokines. Also, BTK plays a role in the activation of the NLRP3, leading to the secretion of IL-1β(8). ***ACE_2_:*** angiotensin converting enzyme 2; ***FcγRs:*** Fc gamma receptors; ***TLR:*** Toll-like receptors; ***NF-ƙB:*** Nuclear factor-κB; ***BTK:*** Bruton Tyrosine Kinase; ***NLRP3:*** NLR Family Pyrin Domain Containing 3; ***BTKi:*** BTK inhibitor; ***IL-6:*** Interleukin 6; ***IFNγ:*** interferon gamma; ***TNF-α:*** Tumour Necrosis Factor alpha.

Bruton’s tyrosine kinase (BTK) is a non-receptor intracellular tyrosine kinase, important in the development of various stages of B lymphocytes. Historically, its mutation initially was described in X-linked agammaglobulinemia (XLA) (8). However, the expression of BTK is not limited to B cells and is expressed by all cells of the hematopoietic lineage except T cells, natural killer (NK) cells, and plasma cells (9, 10). BTK is an essential agent active in innate immunity (11) and has an important role in multiple signaling pathways. In neutrophils, it mediates signaling via Toll-like receptor 4 (TLR-4) (12) and in human macrophages and dendritic cells, BTK is involved in the recognition of pathogens via multiple TLRs (11). In macrophages, TLRs recognize RNA from respiratory viruses, like SARS-CoV-2, and initiate signaling through BTK-dependent activation of nuclear factor ƙB (NF-ƙB). BTK regulates transcription factors, such as NF-ƙB and IFN-regulatory factors, which are important for macrophage M1 polarization leading to the production of multiple inflammatory chemokines, cytokines, and phagocytosis (Figure 1) (8, 13).

Concerning the important role of BTK in cytokine storm and hyperinflammation, it is not surprising that the inhibition of this enzyme can prevent or ameliorate tissue damage in acute lung injury caused by some pathogens. The blockage of BTK may be helpful in the treatment of lethal influenza-induced lung injury in a mouse model (12). Also, Soresina et al. reported two confirmed cases of COVID-19 from Italy that both were known cases of XLA without any B cell in peripheral blood who developed interstitial pneumonia. Both cases could recover without the need for intensive care or oxygen ventilation (14). It may be postulated that the lack of BTK in myeloid cells of XLA patients may have some advantages when they infect with SARS-CoV-2.

Ibrutinib, zanubrutinib, and acalabrutinib are synthetic BTK inhibitors (BTKi) used for the treatment of some hematologic malignancies, like B cell lymphoma and chronic lymphocytic leukemia (CLL). Also, the Food and Drug Administration (FDA) licensed ibrutinib for the treatment of chronic graft versus host disease (cGVHD) (15). Treon et al. recently reported six cases of Waldenstrom macroglobulinemia receiving ibrutinib who experienced COVID-19. Ibrutinib continued at full dose in five cases (420 mg daily) but the sixth case was on a reduced dose (140 mg daily) due to arthralgia. The course of COVID-19 was mild in cases on the recommended dose of ibrutinib. They did not experience dyspnea and there was no need for supplemental oxygen or hospitalization. However, the sixth patient who received a low dose of ibrutinib experienced a severe form of the disease and needed hospitalization and also was unresponsive to hydroxychloroquine and intravenous immunoglobulin. His condition deteriorated and eventually, he required mechanical ventilation. When the dose of ibrutinib increased to 420 mg daily, the patient's condition improved dramatically, and finally, he was extubated and discharged from the hospital. The authors concluded that ibrutinib and possibly other BTKis may be effective in protection from the severe form of COVID-19, or even for an improvement in lung injury caused by this virus (16). In another report Thibaud and colleagues reported eight cases under BTKi (seven received ibrutinib and one acalabrutinib) were hospitalized due to COVID-19. BTKi in six patients was stopped, of whom two cases eventually died of COVID-19 but others experienced the mild to moderate disease. The two patients who continued BTKi, experienced a milder form of COVID-19, shorter course of hospital stay, and finally, completely recovered (15).

Although theoretically and based on the limited experiences mentioned above, BTK inhibitors are promising agents to control COVID-19, to date, there is no confirmed evidence supporting their usage. As our knowledge, four clinical trials registered at the “ClinicalTrials.gov” database on the potential benefit of BTKi in COVID-19 are underway.

On the other hand, some concerns should be considered about the usage of BTKi in COVID-19. The theoretical risk of humoral immunosuppression and opportunistic infections may be important and secondary bacterial and fungal infections, particularly pneumonia are commonly reported among patients under treatment with BTKi (13, 17). A systematic review showed the rate of 56% for infectious complications among patients under ibrutinib as a single agent regimen in the treatment of hematologic malignancies. Approximately 20% of the patients developed pneumonia, many of them due to opportunistic pathogens, and 2% of them died (18). The potential effect of BTKi on the host's ability to develop immunity to a vaccine should also be considered. To date, there is no effective vaccine against SARS-CoV-2, and the available data on the effect of BTKi on vaccine efficacy is limited, but due to the suppressive effect of BTKi on the humoral immune system, seroconversion may be decreased after vaccination (13). If clinical trials confirm the efficacy of BTKi in the treatment of COVI-19, it may arise the question that “Which BTKi is better?” Acalabrutinib as the second-generation BTKi is more selective than ibrutinib with a lower incidence of drug adverse effects.

In conclusion, available data suggest that the immunomodulatory intervention may be the vital key in the treatment of excessive inflammation during COVID-19 and BTK may be a promising drug target to control severe lung injury in this context. In the present time, it would be prudent to wait for the finalization and the results of ongoing clinical trials.
